# Gender, Migration and HIV in Rural KwaZulu-Natal, South Africa

**DOI:** 10.1371/journal.pone.0011539

**Published:** 2010-07-12

**Authors:** Carol S. Camlin, Victoria Hosegood, Marie-Louise Newell, Nuala McGrath, Till Bärnighausen, Rachel C. Snow

**Affiliations:** 1 Center for AIDS Prevention Studies, University of California San Francisco, San Francisco California, United States of America; 2 Department of Epidemiology and Population Health, London School of Hygiene and Tropical Medicine, London, United Kingdom; 3 Africa Centre for Health and Population Studies, Nelson R. Mandela School of Medicine, University of KwaZulu-Natal, Somkhele, South Africa; 4 Centre for Paediatric Epidemiology and Biostatistics and Institute of Child Health, University College London, London, United Kingdom; 5 Department of Global Health and Population, Harvard School of Public Health, Boston, Massachusetts, United States of America; 6 Department of Health Behavior and Health Education, University of Michigan School of Public Health, Ann Arbor, Michigan, United States of America; University of Cape Town, South Africa

## Abstract

**Objectives:**

Research on migration and HIV has largely focused on male migration, often failing to measure HIV risks associated with migration for women. We aimed to establish whether associations between migration and HIV infection differ for women and men, and identify possible mechanisms by which women's migration contributes to their high infection risk.

**Design:**

Data on socio-demographic characteristics, patterns of migration, sexual behavior and HIV infection status were obtained for a population of 11,677 women aged 15–49 and men aged 15–54, resident members of households within a demographic surveillance area participating in HIV surveillance in 2003–04.

**Methods:**

Logistic regression was conducted to examine whether sex and migration were independently associated with HIV infection in three additive effects models, using measures of recent migration, household presence and migration frequency. Multiplicative effects models were fitted to explore whether the risk of HIV associated with migration differed for males and females. Further modeling and simulations explored whether composition or behavioral differences accounted for observed associations.

**Results:**

Relative to non-migrant males, non-migrant females had higher odds of being HIV-positive (adjusted odds ratio [aOR] = 1.72; 95% confidence interval [1.49–1.99]), but odds were higher for female migrants (aOR = 2.55 [2.07–3.13]). Female migrants also had higher odds of infection relative to female non-migrants (aOR = 1.48 [1.23–1.77]). The association between number of sexual partners over the lifetime and HIV infection was modified by both sex and migrant status: For male non-migrants, each additional partner was associated with 3% higher odds of HIV infection (aOR = 1.03 [1.02–1.05]); for male migrants the association between number of partners and HIV infection was non-significant. Each additional partner increased odds of HIV infection by 22% for female non-migrants (aOR = 1.22 [1.12–1.32]) and 46% for female migrants (aOR = 1.46 [1.25–1.69]).

**Conclusions:**

Higher risk sexual behavior *in the context of* migration increased women's likelihood of HIV infection.

## Introduction

A growing literature confirms that migration confers economic benefit to migrants and their households [1], [2], [3], [4], [5], [6], [7], [8], [9], [10], and this is no less true in South Africa, where in the 1990s some 16% of the rural population moved annually to urban areas [11] to escape poverty and provide financial support to families left behind [5], [12], [13]. The negative consequences of migration on the HIV epidemic are well-documented, with ample historic evidence that HIV spread between urban areas, and from urban to rural areas, via corridors of population movement [14], [15], [16], [17]. However, literature on migration and HIV has largely focused on risk to male labor migrants and their non-migrant female partners, or migrants overall [15], [17], [18], [19], [20], [21], [22], [23], [24], [25], [26]. The few studies that have examined the HIV risks to women via their direct participation in migration report higher risk behaviors, and higher HIV prevalence compared to non-migrant women [12], [20], [27], [28], [29], [30], [31].

While statistical comparisons of migrants and non-migrants have been undertaken in male and female populations, to our knowledge no studies have tested the hypothesis of potential sex differences in HIV risks associated with migration. However, a small body of evidence suggests that the risks of HIV via migration may differ for women and men. In South Africa, women tend to migrate shorter distances to informal settlement areas and regional towns, and retain ties to rural homes, while men tend to migrate longer distances to urban areas, and are less likely to return to households of origin [12], [27], [28], [29], [30], [31]. If the typical corridors of migration differ for males and females in a given region, male and female migrants may be exposed to sexual networks and geographic areas with different levels of HIV prevalence and therefore different probabilities of infection with a given sexual act [32], [33]. In the context of South Africa, population-based studies have found HIV prevalence to be up to twice as high in informal settlements and peri-urban areas compared to urban and rural areas [17], [26], [34], [35], [36], [37], [38]. Moreover, migration may result in different “behavioral consequences” for men and women. For example, women tend to access informal sector work (e.g., market vending or beer-brewing) with less exposure to workplace health and prevention programs. Migrant women working in the informal sector may also face pressure to offer sex in exchange for money, commodities, transportation or housing [20], [24], [38], [39]. Having multiple households may foster having multiple “main” lovers - men with whom condom use is least likely [20], [39], [40].

The strikingly higher HIV prevalence for younger women compared to younger men in sub-Saharan Africa [41], [42], coupled with a growing literature documenting women's increasing participation in migration in the region [12], [28], [31], [37], [43], raises the question of how much of this disparity may be attributable, in part, to a differential risk of HIV with migration for males and females. The present study examines whether the risk of HIV associated with migration is similar, or different, for males and females in a well-characterized population in KwaZulu-Natal (KZN), South Africa [44], [45], [46], [47], [48]. Finding that migration confers a higher risk of HIV to women than to men, we explore several possible mechanisms for this difference, including possible differences in the negative behavioral consequences of migration. Finally, we examine whether sex, migration and behavior together predict the likelihood of being infected.

## Methods

### Study design

This is a cross-sectional study of sex differentials in the association between migration and HIV prevalence, using longitudinal data on migration and other socio-demographic chararacteristics in a surveillance population. The analytical dataset used data on migration events and socio-demographic characteristics which were collected prior to the HIV test date.

### Setting and data source

Data are from the Africa Centre Demographic Information System (ACDIS) of the Africa Centre for Health and Population Studies (www.africacentre.com). Since 2000, data on the characteristics of households and individuals living in a 435km^2^ area, including births, deaths and migrations (semi-annually); and measures of socioeconomic position (annually) have been collected in a household-based surveillance in a primarily rural area of KZN. ACDIS was designed to mirror the social dynamics of individuals and households in KZN [48]. Individuals are included in the surveillance population on the basis of being a member of a household in the study area irrespective of whether the person is a resident or not. The residency status and place of residence is routinely recorded for all household members. The place of residence is typically considered to be the homestead where a person keeps their daily belongings and spends most nights. While it is possible for an individual to be considered a *member* of more than one household at a time, for instance in the case of polygamous marriage, an individual can only be recorded as *resident* at one residence at any point in time. At each fieldworker visit, any change in household residency is recorded, together with information about the origin or destination and the date of the move. Changes in residency are referred to as migration events. These are classified as in-migrations (a migration into a homestead within the surveillance area), internal migrations (migrations within the surveillance area) or out-migrations (migration to a homestead outside of the area). Migrations are also described as being an individual or a household migration. A household migration involves a change of residence by all resident members of the household. This study examined individual migrations only.

Annual HIV serological and sexual behavior surveys were implemented in 2003 for all residents [49]. Females aged 15 to 49 and males aged 15 to 54 years were eligible to be included. The dataset also included information on sexual behavior collected in the same round as the HIV surveillance data, and information from a Household Socio-Economic Survey (HSE-2), carried out in 2003 and the first half of 2004. Data were collected via face-to-face interview. (The first round of sexual behavior surveillance also used, in a sub-population, a “secret voting” method which utilized guided self-completion of an answer sheet, for the most sensitive items. The method was tested to ascertain whether it yielded greater level of reporting of higher risk sexual behavior. For these analyses, the data collection “method effect” was examined, and not found to significantly affect the findings.) Ethical approval and methods for ensuring informed consent for participation in ACDIS have been reported previously [48], [49].

### Participants and adjustment for potential selection bias

This analysis used data for 11,677 members of the population who were residents on 01 June 2003, eligible to participate in HIV surveillance, and participated in testing (see Figure S1 for more information about the development of the dataset for analysis.) Contact rates for HIV surveillance were reported previously [49]. There were systematic differences between participants and non-participations in HIV surveillance, but other ACDIS data permitted comparisons of the characteristics of these groups in order to determine, to the extent possible, the direction and strength of selection bias. (The following characteristics were associated with participation in the first round of HIV surveillance: sex; age; whether the individual died before 01 January 2007, or remained alive; partnership status; employment status; education level; tertile of household assets; whether ever internally migrated since the start of ACDIS; whether in-migrated since the start; whether the individual was resident on 01 June 2003; degree of presence in the household in the previous 6 months; whether or not present in the night prior to the visit; and household infrastructural variables related to electricity, access to a flush or chemical toilet and access to a piped water supply. All of these measures were included in the propensity score logit model specification.) We corrected the data for this bias on observable covariates of participation using the Propensity Score weighting approach [50]. In the method, *X_i_* covariates are observed for both respondents and non-respondents; *M* is the missing data (participation in testing) indicator (where non-respondent = 0 and respondent = 1). The propensity score specification is estimated using a logit model, i.e.:
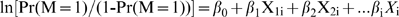
Where *X_i_* … represents the covariates of testing. The predicted probabilities from this model are the propensity scores. A weight was generated by dividing the mean HIV test participation rate by the predicted probabilities, i.e. weight = r(mean tested)/Pr(M = 1). This propensity score weight was then used as a non-response adjustment weight.

### Variables

The principal outcome variable was HIV infection status (HIV antibody-positive or -negative). The key independent variables for analysis were sex [51] and migration status. This study examined the three types of individual migration recorded in ACDIS, individual in-migration to the surveillance area, internal migration within it, and out-migration from it. We examined only the migrations which preceded HIV surveillance (i.e., prior to the HIV surveillance visit date, or 01 June 2003 for non-participants in HIV surveillance.) We constructed a dichotomous measure of any migration of any type within the two years prior to the HIV surveillance visit date (or 01 June 2003, for non-participants) vs. none; we also used a categorical measure of none, 1 and 2 or more migrations since the start of ACDIS (01 January 2000). ACDIS also records the number of nights present in the household in the six months prior to the most recent visit for all members of households. We examined the recent mobility of residents using this measure, categorized into three levels: at home every night, most nights, or approximately half or fewer nights in six months prior to the HIV surveillance visit. Measures of sexual behavior associated with HIV infection included: the numbers of sexual partners in the lifetime, past year and concurrently (these data previously reported in Todd *et al.*, 2009 [52]); ever use of a condom; perceived personal risk of HIV infection in the past or present; previously received counseling and testing (VCT) for HIV; and experience of any sexually transmitted infection (STI) symptoms (abnormal discharge or genital ulcer) in the past year (these data previously reported in White *et al.*, 2008 [53]).

On the basis of prior research and the data available to this study, we posited that a “risk predisposition” for both migration and HIV infection will be associated with age, employment status, education level, marital/partnership status, and measures of household socio-economic status (using infrastructure variables and tertiles of the number of assets). These were treated as potential confounders in modeling. We also included, as a control variable, whether or not the individual experienced the loss of another adult in his or her household to AIDS in the period between the start of ACDIS and 01 June 2003, which may have facilitated migration and could potentially be a marker of HIV infection [54], [55].

### Statistical analysis

For characteristics defined as continuous variables, we used the Wilcoxon-Mann-Whitney test to compare migrants and non-migrants in the populations of resident men and women, respectively; chi-square tests were used to test group differences for categorically-defined characteristics. Additive and multiplicative effects logistic regression models [56] were fitted in a sequence following a set of hypotheses, all of which used prevalent HIV infection status as dependent variable. Independent variables for the models were selected on the basis of theoretical significance from prior research and their significant association with prevalent infection in simple logistic regression models. We also used ‘G-computation’, a structural equation modeling approach [57], to model two theoretical outcomes, in order to examine the possible influence of the sex composition of the population of migrants on observed findings. Missing data on the characteristics and sexual behaviors associated with HIV prevalence were accommodated in the analysis (retained and coded as missing for categorical variables and imputed for continuous variables.) The significance level of 95% was used for all tests. Analyses were carried out using Stata version 10 software [58].

## Results

**Figure 1:**
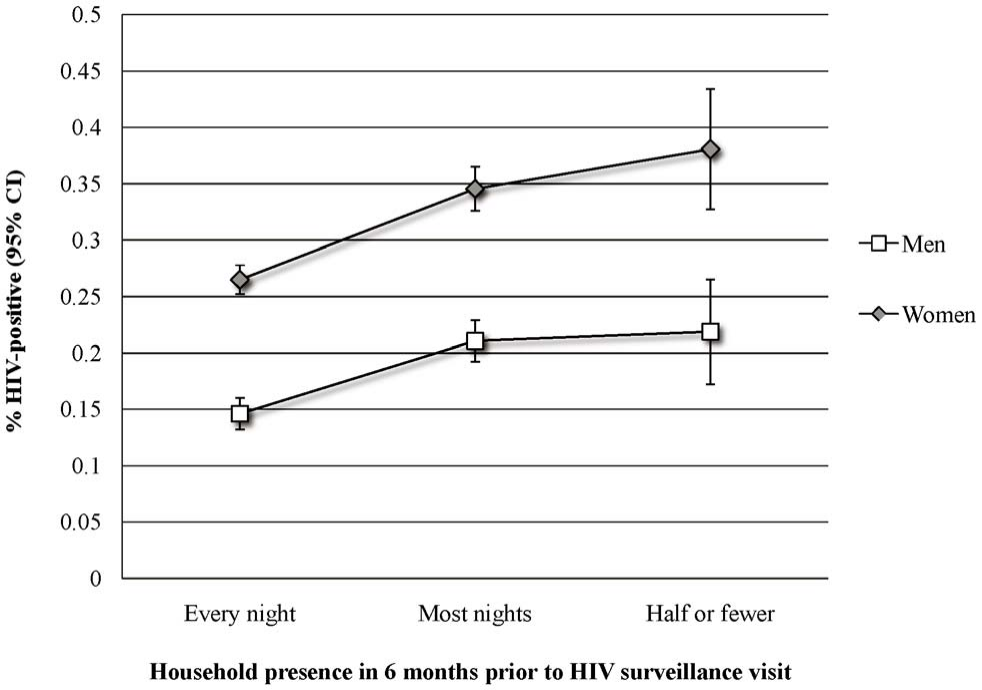
Prevalent HIV infection among resident household members by sex and a measure of recent mobility. Data are for population of residents eligible for HIV testing on 01 June 2003 (n = 11,677). Propensity score weight applied.

The population was highly mobile: 35.8% of men (95% CI, 34.5–37.1%) and 38.0% of women (95% CI, 36.8–39.1%) migrated at least once in the two years prior to HIV surveillance. (Of note, persons who out-migrated before 1 June 2003 and did not retain household membership are not included; men were more likely to out-migrate and not return.) In non-migrants, HIV prevalence was higher in women (29.5%; 95% CI, 28.3–30.7%) than in men (18.9%; 95% CI, 17.6–20.1%); the sex disparity was wider in those who had migrated: among migrants, 42.8% of women (95% CI, 40.5–45.0%) vs. 24.4% of men (95% CI, 21.9–26.9%) were HIV antibody-positive. Figure 1 shows a dose-response relationship between degree of absence from the household and HIV prevalence among residents, higher at every level and more steadily positive for women. For resident women who spent half or fewer nights in the household in the previous six months, HIV prevalence peaked at 38.1% (5% CI, 33.4–42.7%). Among resident men, prevalence levels did not vary by degree of mobility beyond the first level of presence in the household (every night).

Table 1 shows socio-economic characteristics of resident men and women and associations with HIV prevalence (odds ratios adjusted for age and age^2^). HIV prevalence was highest in the 25 to 34 year age group, in which 37.8% of men and 48.4% of women were found to be HIV-positive. A wide sex disparity was evident in those aged 15 to 24, among whom 19.1% of women and 4.0% of men were HIV-positive. For both men and women, prevalence was highest among those with a regular, non-marital partner, relative to other marital status categories. Prevalence levels did not significantly differ across employment status or education level categories (with the exception of significantly lower age-adjusted odds of infection among current full-time students than among those with little or no education.) Prevalence levels were higher among resident members of households with access to electricity compared to those without, and were higher for resident women in households with access to piped water and sanitation. Also among resident women only, prevalence was significantly higher among those with households in which another adult died of AIDS since January 2000.

**Table 1:** Selected socio-economic and behavioral characteristics of the resident population by HIV infection status.

	Men					Women				
Characteristic	N	% HIV+	Adj. OR	95% CI		N	% HIV+	Adj. OR	95% CI	
**Age group (10-year)** **										
15–24	2,979	4.0	1.00			3,443	19.1	1.00		
25–34	723	37.8	**11.87**	**8.93**	**15.78**	1,365	48.4	**3.44**	**2.87**	**4.12**
35–44	545	30.8	**9.73**	**7.04**	**13.44**	1,440	29.1	**1.47**	**1.23**	**1.75**
45–54 men/45–49 women†	520	19.4	**5.88**	**3.88**	**8.90**	662	20.5	0.96	0.76	1.21
**Partnership pattern**										
Marital partner	432	19.4	1.00			1,159	18.6	1.00		
No current partner	1,559	7.7	**1.78**	**1.19**	**2.66**	1,114	29.6	**2.90**	**2.20**	**3.82**
Non-marital partner††	1,535	28.2	**2.97**	**2.09**	**4.24**	3,313	38.4	**2.86**	**2.28**	**3.58**
Missing	134	8.2	**5.09**	**1.99**	**13.04**	169	27.2	**2.08**	**1.31**	**3.30**
Not yet started sex	1,067	0.8	0.37	0.17	0.82	1,255	3.8	**0.38**	**0.26**	**0.57**
**Employment**										
No earned income	2,159	15.1	1.00			3,757	31.1	1.00		
Does something to earn money	956	28.9	1.18	0.91	1.53	1,393	34.7	**1.09**	**0.90**	**1.32**
Refused, missing or NA	1,652	3.5	1.34	0.20	2.11	1,760	12.6	0.89	0.67	1.17
**Education level**										
None through Standard 5	1,257	24.9	1.00			2,344	31.0	1.00		
Standard 6 to 9	756	23.2	1.03	0.79	1.33	1,496	37.5	1.06	0.88	1.27
Standard 10/Matric or more	495	20.4	0.80	0.58	1.12	811	33.9	0.83	0.66	1.27
Full-time student	2,056	1.4	**0.26**	**0.15**	**0.47**	1981	11.0	**0.44**	**0.33**	**0.58**
Missing	203	20.7	1.41	0.84	2.36	278	33.5	1.08	0.75	1.57
**Household infrastructure**										
No electricity	2,201	11.0	1.00			3,404	23.1	1.00		
Has electricity source	2,322	16.0	**1.60**	**1.26**	**2.03**	3,163	30.8	**1.50**	**1.30**	**1.73**
Missing	244	19.3	**2.28**	**1.40**	**3.71**	343	32.4	**1.54**	**1.08**	**2.20**
Other water source	2,295	12.6	1.00			3,327	25.0	1.00		
Piped water (private/public)	2,260	14.6	1.03	0.81	1.29	3,292	28.7	**1.23**	**1.07**	**1.42**
Missing	212	19.8	1.58	0.94	2.66	291	33.3	1.43	0.98	2.07
No flush or chemical toilet	3,236	13.2	1.00			4,774	25.4	1.00		
Flush toilet/ventilated pit	1,315	14.3	0.96	0.75	1.21	1,840	30.7	**1.37**	**1.17**	**1.60**
Missing	216	20.4	**1.70**	**1.04**	**2.76**	296	33.5	1.42	0.98	2.05
No prior adult AIDS death	3,957	13.4	1.00			5,719	25.6	1.00		
≥1 other adult member died of AIDS	810	16.2	1.18	0.90	1.55	1,191	34.2	**1.51**	**1.24**	**1.85**

Table 1 note: Row percentages shown, separately for men and women, respectively; percentages are weighted and frequencies are unweighted. Weights are propensity score weights based upon probability of participation in HIV testing. Statistically significant group differences (at *p*<.05) are highlighted in bold-print. Odds ratios are adjusted for age and age^2^ (due to a non-linear association between age and HIV status).

*Data are from the resident population who were eligible for testing on 01 June 2003 and not included in the non-resident sample selected for participation in HIV testing at that time. N = 11,677.

**The odds ratios given for the model regressing HIV test result on 10-year age group by sex has only one independent variable (i.e. unlike the other models shown in this table, single year of age and age^2^ not included as additional variables.).

†Women only up to age 49 were eligible for HIV testing; men up to age 54 were eligible.

††This category mainly comprised of those with a regular non-marital partner: a small minority (n = 407) reported a casual partner. HIV prevalence was lower among those reporting a casual partner (14.0%) than among those with a regular non-marital partner (37.1%), and closer to the level of those with a marital partner (18.8%).

Table 2 shows reported sexual behaviors by past two-year migration status and HIV prevalence among residents. In summary, men reported a higher number of sexual partners in the past year than did women, were more likely to have used condoms, and less likely to have received VCT. Compared to non-migrants, migrants reported more lifetime partners (6.4 vs. 6.2 in men, marginally significant at *p* = 0.059; and 2.1 vs. 1.9 in women, *p* = <.0001) and past year partners (1.9 vs. 1.6 in men, *p* = <.0001; and 0.78 vs. 0.83 in women, *p* = 0.012). Male migrants were significantly more likely than male non-migrants to have experienced STI symptoms in the past year, and migrants of both sexes were significantly more likely than their non-migrant counterparts to have ever used a condom, feel that they were at risk of HIV, and have previously received VCT.

**Table 2:** Sexual behavior, HIV risk perception, prior VCT and STI symptoms among men and women, by migration status.

	MEN			WOMEN		
Reported sexual behavior	Migrants^a^ (n = 1,432)	Non-migrants (n = 4,469)		Migrants^a^ (n = 3,297)	Non-migrants (n = 7,996)	
	Mean ± SDMedian (IQR)	Mean ± SDMedian (IQR)	p	Mean ± SDMedian (IQR)	Mean ± SDMedian (IQR)	p
Lifetime number of sexual partners	6.4±7.7	6.2±7.5	0.059	2.1±1.4	1.9±1.3	<.0001
	5 (2-8)	4 (2-8)		2 (1-3)	2 (1-2)	
Past year number of sexual partners	1.9±1.9	1.6±1.8	<.0001	0.78±0.6	0.83±0.5	0.012
	1 (1-2)	1 (1-2)		1 (1-1)	1 (0-1)	
Concurrent partnerships	1.3±1.4	1.2±1.2	0.120	0.77±0.5	0.79±0.5	0.120
	1 (1-2)	1 (1-1)		1 (1-1)	1 (0-1)	

	N	%	N	%		N	%	N	%	
Ever used a condom	574	40.1	1,334	29.9	0.001	470	14.3	842	10.5	<.0001
Never used a condom	507	35.4	1,507	33.7		1,454	44.1	3,444	43.1	
Missing	351	24.5	1,628	36.4		1,373	41.6	3,710	46.4	
Perceives self to be at high risk of HIV^b^	318	22.2	779	17.4	<.0001	646	19.6	1,400	17.5	0.016
Does not perceive self to be at risk	1,060	74.0	3,505	78.4		2,478	75.2	6,103	76.3	
Missing	54	3.8	185	4.1		173	5.3	493	6.2	
Ever received voluntary counseling & testing for HIV (prior to visit)	245	17.1	590	13.2	<.0001	855	25.9	1,592	19.9	<.0001
Never previously received HIV-VCT	1,174	82.0	3,830	85.7		2,416	73.3	6,342	79.3	
Missing	13	0.9	49	1.1		26	0.8	62	0.8	
Had abnormal discharge or genital ulcer in past year	125	8.7	276	6.2	<.0001	507	15.4	1,128	14.1	0.203
No STI symptom in past year	658	46.0	1,881	42.1		1,309	39.7	3,193	39.9	
Missing	649	45.3	2,312	51.7		1,481	44.9	3,675	46.0	

Table 2 notes: Data shown are shown for the total population eligible for HIV testing on 01 June 2003 who participated in the sexual behavior surveys (n = 5,901 men and n = 11,293 women.) For continuous variables, two-sided differences in distributions of characteristics between migrants and non-migrants, in men and in women, tested with the Wilcoxon rank sum test. For categorical variables, the chi-square test was used (excluding ‘missing’ category).

a Migration in Table 2 defined as at least one change of residence as an individual (in-migration, out-migration or internal migration) in the two years prior to the HIV test or eligibility date (June 1, 2003) for individuals who did not participate in testing.

b Respondent agrees with either of the following questions: “is there anything that happened to you in the past that may have put you at risk of becoming infected with HIV?” or “are you currently in a situation where you may be at risk of becoming infected with HIV?”.

Table 3 shows multiple logistic regression models for HIV infection (Model 1) in the resident population. Three measures of migration were examined: any vs. no migration in the past two years (1A), number of migrations in the past two years (1B) and a measure of the continuum of recent presence in the household (1C). All were independently associated with HIV: those who had migrated in the past two years had a 29% higher odds of being HIV-positive in 2003–04 relative to those with stable residence (A) (aOR 1.29; 95% CI, 1.11–1.5); each migration in the past two years conferred 25% higher odds of being HIV-positive (B) (aOR 1.25; 95% CI, 1.09–1.43); and, relative to those present “every night”, those present “most nights” had 18% higher odds (aOR 1.18; 95% CI 1.06–1.32), and those present “half or fewer of the nights” had 53% higher odds of HIV infection (aOR 1.15; 95% CI, 1.15–2.03). The other covariates, with patterns of association with HIV prevalence similar to those reported in Table 1, did not differ markedly across the models. In summary, analyses shown in Table 3 confirmed that the odds of being HIV-infected were higher among resident women than men, net of the effects of other factors, and the odds of infection were higher among those who migrated relative to those who did not. Each step increase in the degree of absence from the rural household was associated with higher odds of infection among residents.

**Table 3:** Multiple logistic regression models of HIV infection risk (Models 1.A–C).

HIV test result (1 = positive)	1) HIV = SEX+MIGRATION								
	A) MIGRATION IN PAST 2 YEARS			B) FREQUENCY OF MIGRATION			C) MOBILITY IN PAST 6 MONTHS		
	aOR	95% CI		aOR	95% CI		aOR	95% CI	
**A) Migrated in past 2 years**									
*Stable residence in past 2 years*	1.00								
Any migration (in-, out- or internal)	1.29^***^	1.11	1.50	–	–	–	–	–	–
**B) Sum of migrations in past 2 years**	–	–	–	1.25^***^	1.09	1.43	–	–	–
**C) Household presence, past 6 months**									
*Every night*							1.00		
Most nights	–	–	–	–	–	–	1.18^**^	1.06	1.32
Approximately half or fewer nights	–	–	–	–	–	–	1.53^**^	1.15	2.03
**Sex** *Male*	1.00								
Female	1.96^****^	1.70	2.25	1.95^****^	1.70	2.24	1.95^****^	1.70	2.24
**Age group** *15–24*	1.00								
25–34	2.75^****^	2.29	3.30	2.79^****^	2.33	3.35	2.81^****^	2.33	3.39
35–44	1.75^****^	1.41	2.17	1.78^****^	1.43	2.21	1.78^****^	1.43	2.21
45–54 men/45–49 women	1.37^*^	1.02	1.84	1.39^*^	1.03	1.87	1.37^*^	1.02	1.85
**Education level** *0 - Standard 5*	1.00								
Standard 6 to 9	1.03	0.88	1.21	1.04	0.89	1.21	1.03	0.88	1.21
Standard 10 (Matric) or higher	0.75^***^	0.61	0.93	0.75^**^	0.61	0.93	0.75^***^	0.61	0.93
Full-time student	0.42^****^	0.33	0.54	0.43^****^	0.33	0.55	0.42^****^	0.33	0.55
Missing	1.65	0.98	2.78	1.64	0.97	2.78	1.61	0.95	2.73
**Employment** *No earned income*	1.00								
Does something to earn money	1.16	0.99	1.35	1.17^*^	1.00	1.36	1.16^*^	0.99	1.35
Refused, missing or NA	0.47^****^	0.36	0.60	0.47^****^	0.37	0.61	0.47^****^	0.36	0.60
**Household infrastructure** *No electricity*	1.00								
Has electricity source	1.55^****^	1.36	1.77	1.56^****^	1.37	1.78	1.52^****^	1.33	1.73
Missing	1.76^**^	1.14	2.70	1.77^**^	1.15	2.72	1.77^*^	1.14	2.76
**Partnership pattern** *Marital partner*	1.00			1.00			1.0		
No current partner	1.58^****^	1.26	1.98	1.59^****^	1.26	1.99	1.62^****^	1.29	2.04
Non-marital partner	2.84^****^	2.34	3.43	2.84^****^	2.35	3.44	2.87^****^	2.37	3.47
Missing	2.15^**^	1.20	3.85	2.17^**^	1.22	3.87	2.20^***^	1.28	3.79
*No prior adult AIDS death in household*	1.00			1.00			1.00		
≥1 other adult died of AIDS, 2001–'03	1.36^****^	1.16	1.60	1.35^****^	1.15	1.59	1.35^****^	1.15	1.59

Table 3 notes: Propensity score weighting applied. Data are from the resident population who were eligible for testing on 01 June 2003 and who were not included in the non-resident sample selected for participation in HIV testing at that time. N = 11,677. Wald χ^2^ *(df)* for Model 1.A = 1206.2 *(17)*, Model 1.B = 1205.7 *(17)*, Model 1.C = 1,224.2 *(19)*.

^*^ *p*<.05; ^**^ *p*<.01; ^***^ *p*<.001; ^****^ *p*<.0001.

In Table 4, first column, Model 2 tested the hypothesis that the odds of HIV infection were higher for female migrants than for male migrants, and higher for female migrants than for non-migrants of both sexes, by including the interaction term sex *x* migration and the above covariates. (The models shown in Table 4 used the dichotomous measure of past two-year migration). For men, having recently migrated was not significantly associated with being HIV-positive. Female non-migrants had 72% higher odds of infection compared to male non-migrants (aOR 1.72; 95% CI 1.49–1.99), and female migrants had more than double the odds of infection (aOR = 2.55; 95% CI 2.07–3.13) compared to male non-migrants. Female migrants also had 48% higher odds of being HIV-positive than female non-migrants (aOR 1.48, 95% CI 1.23–1.77, not shown). Findings confirmed the hypothesis that sex modifies the association between migration and being HIV-positive: migration was associated with higher risk for women.

**Table 4:** Multiple logistic regression models of HIV infection risk (Model 2–4).

HIV test result (1 = positive)	2) HIV = SEX * MIGRATION			3) HIV = SEX+MIGRATION+BEHAVIORAL RISK			4) HIV = SEX* BEHAVIORAL RISK*MIGRATION		
	aOR	95% CI		aOR	95% CI		aOR	95% CI	
*Male (non-migrant)*	1.00			1.00					
Female (non-migrant)	1.72^****^	1.49	1.99	2.36^****^	2.01	2.76	–	–	–
Male: Migrated in past 2 years	1.01	0.76	1.33	–	–	–	–	–	–
Female: Migrated in past 2 years	2.55^****^	2.07	3.13	–	–	–	–	–	–
*Stable residence in past 2 years*				1.00					
Any migration (in-, out- or internal)	–	–	–	1.27^**^	1.09	1.48	–	–	–
Lifetime no. of sexual partners^a^	–	–	–	1.04^****^	1.02	1.06	–	–	–
Male Non-migrant * Partner number	–	–	–	–	–	–	1.03^****^	1.02	1.05
Male Migrant * Partner number	–	–	–	–	–	–	1.01	0.97	1.06
Female Non-migrant * Partner number	–	–	–	–	–	–	1.22^****^	1.12	1.32
Female Migrant * Partner number	–	–	–	–	–	–	1.46^****^	1.25	1.69
*Does not perceive self to be at risk of HIV*				1.00			1.00		
Perceives self to be at risk of HIV	–	–	–	1.35^***^	1.12	1.63	1.35^**^	1.12	1.61
Missing	–	–	–	1.44^***^	1.22	1.69	1.39^***^	1.18	1.63
*Abnormal discharge or genital ulcer, past year*	–	–	–	1.00			1.00		
No STI symptom in past year	–	–	–	1.28^*^	1.04	1.58	1.25^*^	1.01	1.53
Missing	–	–	–	0.88	0.75	1.04	0.90	0.77	1.06
*Age group: 15–24*	1.00			1.00			1.00		
25–34	2.75^****^	2.29	3.30	2.58^****^	2.14	3.10	2.51^****^	2.09	3.01
35–44	1.77^****^	1.43	2.20	1.61^****^	1.29	2.01	1.55^****^	1.24	1.94
45–54 men/45–49 women	1.37^*^	1.02	1.84	1.24	0.92	1.67	1.19	0.88	1.61
*Education level: None - Standard 5*	1.00			1.00			1.00		
Standard 6 to 9	1.04	0.89	1.21	1.05	0.89	1.22	1.05	0.90	1.23
Standard 10 (Matric) or higher	0.76^***^	0.61	0.93	0.74^***^	0.59	0.91	0.74^***^	0.60	0.92
Full-time student	0.42^****^	0.33	0.54	0.4^****^	0.34	0.57	0.46^****^	0.36	0.59
Missing	1.66	0.98	2.79	1.62	0.97	2.73	1.59	0.95	2.69
*Employment: No earned income*	1.00			1.00			1.00		
Does something to earn money	1.16^*^	1.00	1.35	1.17	0.99	1.36	1.17^*^	1.01	1.37
Refused, missing or NA	0.47^****^	0.36	0.60	0.49^****^	0.38	0.63	0.51^****^	0.40	0.66
*Household infrastructure: No electricity*	1.00			1.00			1.00		
Has electricity source	1.55^****^	1.36	1.76	1.59^****^	1.39	1.81	1.54^****^	1.35	1.76
Missing	1.75^**^	1.14	2.68	1.74^*^	1.14	2.66	1.69^**^	1.11	2.59
*Partnership pattern: Marital partner*	1.00			1.00			1.00		
No current partner	1.57^****^	1.25	1.97	1.66^****^	1.31	2.09	1.61^****^	1.28	2.03
Non-marital partner	2.82^***^	2.33	3.41	2.77^****^	2.29	3.3	2.62^****^	2.12	3.19
Missing	2.14^**^	1.21	3.81	2.25^**^	1.24	4.06	2.09^*^	1.16	3.79
*No prior adult AIDS death in household*	1.00			1.00			1.00		
≥1 other adult died of AIDS, 2001–'03	1.35^****^	1.16	1.59	1.34^****^	1.14	1.58	1.32^***^	1.13	1.55

Table 4 notes: Propensity score weight applied. Data are for population as described for Table 3. Wald χ^2^ *(df)* for Model 2 = 1,214.71 *(18)*, Model 3 = 1259.99(22), Model 4 = 1307.69*(26)*.

a Missing data on number of sexual partnerships over the lifetime were imputed using the means of non-missing cases in the male and female populations of surveillance-eligible residents.

^*^ *p*<.05; ^**^ *p*<.01; ^***^ *p*<.001; ^****^ *p*<.0001.

To test the hypothesis that the sex composition of recent migrants could account for this finding, we applied the ‘G-computation’ estimation method. Under the assumption of no unmeasured confounders and a correctly-specified model, we modeled two theoretical outcomes or “counterfactuals” each for men and women: 1) the adjusted predicted HIV prevalence if all were constrained to be non-migrants, and 2) if all were constrained to be migrants. For men, recent migration appeared to have minimal effect on predicted HIV prevalence: 13.9% if all were migrants (95% CI, 11.0–17.4%) vs. 13.7% if all non-migrant (95% CI, 12.2–15.5%). However, migration was associated with substantially higher predicted HIV prevalence for women: 34.3% if all were migrants (95% CI, 30.8–37.9%) vs. 27.2% if all non-migrant (95% CI 25.6–28.8%). Results indicate little evidence that the sex composition of recent migrants accounted for findings observed in Model 2.

We further tested hypotheses that the behavioral consequences of the decision to migrate vary by sex. As shown in Table 2, men reported significantly more sexual partners than did women within migrants and non-migrant populations, respectively. Female migrants did not report higher risk behavior than male migrants, although they reported higher risk behavior than female non-migrants. The possibility remained, however, that a given level of sexual risk behavior could pose a greater hazard of HIV infection to female migrants than to male migrants if female migrants travel to higher prevalence destinations, are exposed to higher-risk sexual networks, or if some other unmeasured aspect of the migration experience rendered its consequences more hazardous for them. Model 3 (Table 4, second column) was fitted to test the hypothesis that sex, migration and higher risk sexual behavior independently predict HIV infection. A measure of the number of sexual partners over the lifetime was included in the model as a marker of sexual risk behavior. As shown, women had 2.36 times the odds of being HIV-positive compared to men (aOR 2.36; 95% CI, 2.01–2.76), and those who had migrated had 27% higher odds of being HIV-infected (aOR 1.27; 95% CI, 1.09–1.48). Each additional lifetime partner was associated with a 4% increase (aOR 1.04; 95% CI 1.02–1.06). In a model (not shown) with the sexual risk behavior variable added to Model 2, the interaction term of sex *x* migration did not lose significance, again showing that migration was associated with a higher likelihood of being HIV-positive in women than in men.

Model 4 (Table 4, third column) tested the three-way interaction of sex *x* migration *x* behavioral risk for the prediction of HIV infection. This model was fitted to test the hypothesis that migration and sexual behavior were inter-related in the prediction of different levels of HIV risk for men and women. For male non-migrants, each additional lifetime partner was associated with a 3% increase in odds of infection (aOR 1.03; 95% CI, 1.02–1.05). For male migrants, the number of partners was not a significant predictor of HIV infection. Moreover, there was no substantial difference between male migrants and non-migrants in the association of each additional partner with the odds of infection. For female non-migrants, each additional lifetime partner was associated with a 22% increase in odds of infection (aOR 1.22; 95% CI, 1.12–1.32), and for female migrants each additional partner was associated with a 46% increase in odds of infection (aOR 1.46; 95% CI 1.25–1.69). Therefore, the association between higher risk sexual behavior and presence of HIV infection was modified both by sex and by participation in migration. Similar results were obtained using the number of sexual partners in the past year.

## Discussion

Our study found that the lifetime number of sexual partnerships was associated with a higher likelihood of being HIV-positive among female migrants, compared to their non-migrant counterparts and to male migrants and non-migrants. These findings suggest that the consequences of migration for HIV risk are particularly disadvantageous to women: higher risk behavior *in the context of* migration may place women at higher risk than men of acquiring HIV. These results underscore that women in the region are not static, passive recipients of HIV infection from male migrants: they are fully participating in migration, and unfortunately bearing more of the burden of HIV associated with migration. The association between migration and HIV infection in women may be synergistic: migration is associated with a higher likelihood of infection in women than in men, and the number of sexual partnerships increases likelihood of being HIV-positive for migrating women to a greater extent than for migrating men.

High levels of mobility of both men and women may contribute to the sustained high HIV prevalence in the region of southern Africa. Frequent migrants may be important links to geographically-spread sexual networks, and high female mobility may be a factor enabling greater inter-connectedness of sexual networks beyond those created by male migrants alone, potentially contributing to the region's exceptionally high and sustained HIV prevalence. The greater the inter-connectedness among sexual networks, the more quickly and broadly HIV will circulate [32]. Our study highlights the particularly high level of HIV prevalence among female migrants, who, in a context of declining marriage [59], [60], [61] and increasing unemployment [28], [62], [63], comprise a large and possibly increasing proportion of adult women in KZN.

We recognize and did our best to address potential limitations of our study. One concern is a reversal of cause and effect, that is, if those who are infected may be more likely to out-migrate for care and/or to return home to receive care [55]. To examine this problem, we first fitted the main models with those who died in the period after the HIV test included in the analysis. Next, we fitted the same models excluding these individuals. Had the sign of the coefficient for migration been reversed in the latter instance, this would have been an indicator of endogenity of migration to HIV. However, we observed no change in the direction of the estimates, and the values of the coefficients for migration were very slightly higher. This finding provided some evidence in support of our premise that exposures to HIV are heightened for individuals who migrate, especially women.

We recognize social desirability bias may affect sexual behavior measures differently for men and women due to the gendered social norms [64], [65]. While this issue may be serious for epidemiological studies aimed at gauging absolute levels of risk behavior within a population, our study was primarily concerned with comparing migrant vs. non-migrant group differences in behaviors. That is, we assume that any bias in reported behavior due to sex differences in reporting would apply equally to migrants and non-migrants. The incomplete data and systematic item non-response in ACDIS data on sexual behavior may challenge validity. To examine this issue, we fitted the models shown in Table 4 using only non-missing data on sexual partners (excluding cases with missing data rather than imputing their values for this variable). We then compared the findings with the results obtained with imputed values. The results were similar. For example, in model 4, with missing data excluded, the odds of infection increase by just over 30% for each additional lifetime partner, among female non-migrants (aOR 1.31; 95% CI, 1.20–1.43) and by over 60% among female migrants (aOR 1.62; 95% CI 1.36–1.92). The findings presented in this manuscript are if anything slightly more conservative in their estimation of the association between migration status and HIV infection modified by sex and number of sexual partners over the lifetime, suggesting that the findings are robust despite incomplete data for this variable.

We anticipated the potential for sample selection bias in HIV prevalence due to non-random participation in HIV testing and attempted to correct for this bias on the basis of the observable covariates of testing using Propensity Score weighting. A limitation of this cross-sectional study was that it was not possible to know whether the independent variables, chiefly migration, preceded HIV infection. Finally, some risk factors for HIV that may also be associated with migration, such as gender-based violence and transactional sex, could not be included in our analysis because ACDIS does not include measures of these factors in its surveillance. Whether the association between migration and HIV in women is mediated by transactional sex has not yet received adequate attention in HIV prevention research in South Africa; the relationship between gender-based violence and migration (as either a driver or a consequence of migration for women) is also a fertile area for future research.

These limitations notwithstanding, our study points to the overlooked role that women's involvement in migration may play in the enormous epidemic seen in KZN. Findings may support the notion that female migrants may travel to ‘higher risk environments’, destinations higher in prevalence than the common destinations of male migrants, where a given act of unprotected intercourse is more likely to result in infection. Further research will be required, however, to directly test this hypothesis, and to explore alternative explanations for our findings. Detailed studies are also needed to determine the extent to which female migration contributes to HIV epidemics elsewhere in sub-Saharan Africa, where populations are mobile, rapid urbanization is underway, and the burden of HIV risk is borne disproportionately by young women [42]. Research is needed to elucidate factors including the spatial and social features of the main destinations of female migrants that render migration particularly hazardous for women, in order to inform the development of effective HIV prevention interventions for female migrants. Further research should also explore sex differences in reasons for and circumstances that drive migration, and ascertain whether HIV risk varies by the type of migration undertaken. As female migration has become an essential household livelihood strategy in KZN and throughout the region, stepped-up HIV prevention and care efforts are urgently needed to preserve the beneficial aspects of migration for women and their families, and to stave off its most dire consequence.

## Supporting Information

Figure S1 Dataset development.
(0.55 MB TIF)
