# Supplementary figures and images for: Gender, Migration and HIV in Rural KwaZulu-Natal, South Africa
Source: PLoS One. 2010 Jul 12;5(7):e11539. doi: 10.1371/journal.pone.0011539 (PMC2902532; doi:10.1371/journal.pone.0011539)

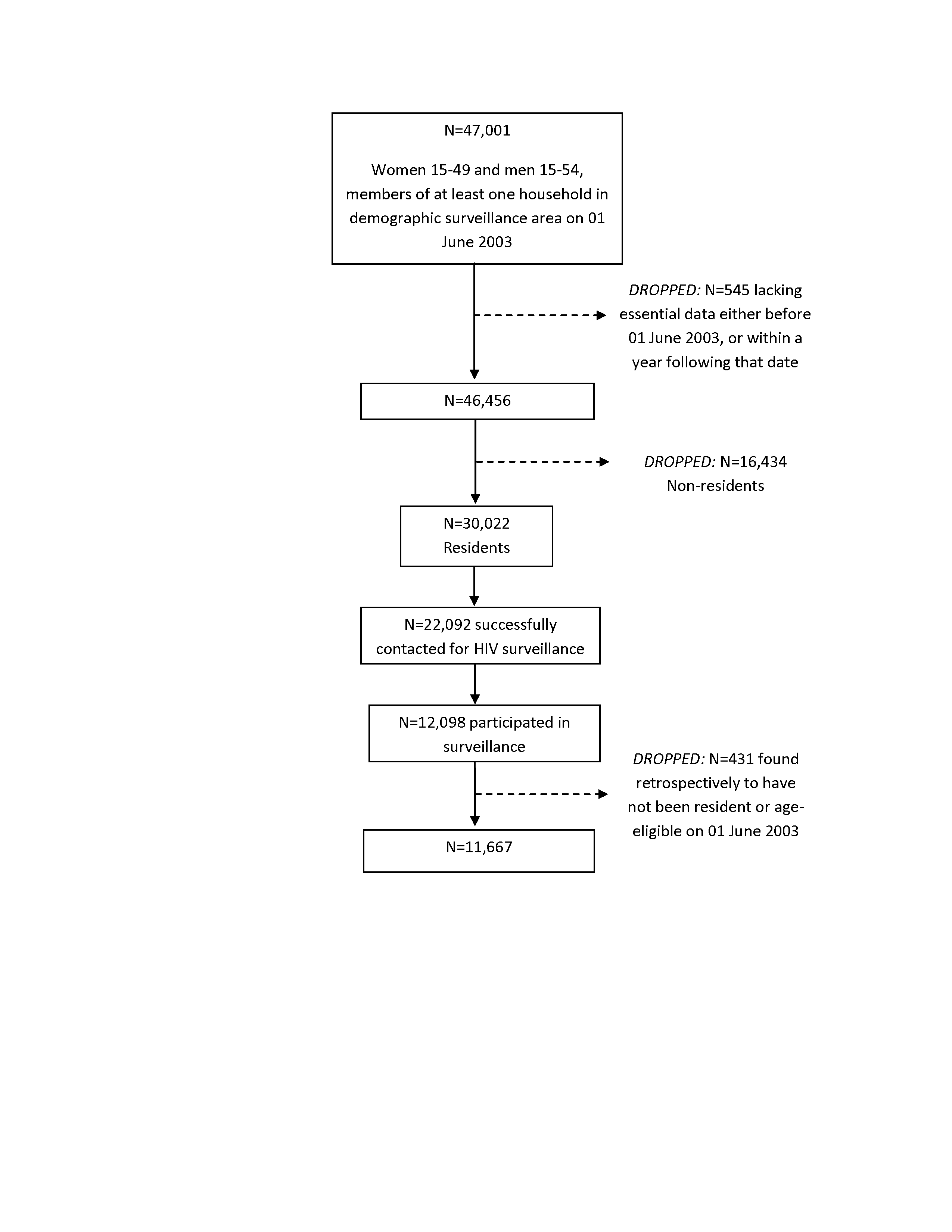

Supplement: Figure S1 — Dataset development. (0.55 MB TIF) [file pone.0011539.s001.tif]
